# An innovative 2D optical navigation workflow for percutaneous pedicle screw fixation in thoracolumbar fractures: comparison with O-arm 3D navigation

**DOI:** 10.3389/fsurg.2026.1801856

**Published:** 2026-06-26

**Authors:** Ren Gao, Kangheng Niu, Le Cheng, Zhong Wei, Xuepeng Wang, Tanjun Wei, Biwang Huang, Feng Xu, Chengjie Xiong

**Affiliations:** 1 Department of Orthopedics, General Hospital of Central Theater Command of the PLA of China, Wuhan, Hubei, China; 2 The First School of Clinical Medicine, Southern Medical University, Guangzhou, Guangdong, China

**Keywords:** 2D optical navigation, learning curve, percutaneous pedicle screw fixation, radiation dose, thoracolumbar fractures

## Abstract

**Objective:**

Thoracolumbar fractures (TLFs) are common spinal injuries caused by high-energy trauma, falls, or osteoporosis. Traditional open surgery may involve substantial blood loss, tissue damage, and prolonged recovery. Percutaneous pedicle screw fixation (PPSF) is minimally invasive and can accelerate rehabilitation, but it often depends on repeated fluoroscopy, increasing radiation exposure and potential neurovascular risk. This study compared the clinical outcomes of two-dimensional (2D) optical navigation and three-dimensional (3D) navigation systems for PPSF in TLFs, focusing on operative time, radiation dose, screw placement accuracy, clinical recovery, complications, and learning curves.

**Methods:**

We retrospectively analyzed 105 patients with TLFs who underwent PPSF at our center from January 2020 to February 2024. Patients were divided into a 2D-navigation group (PPSF assisted by a 2D optical navigation system) and a 3D-navigation group (PPSF guided by a 3D navigation system). Intraoperative and postoperative outcomes—including operative time, radiation dose, pedicle screw accuracy, and clinical recovery assessed by visual analog scale (VAS) and Oswestry Disability Index (ODI)—were compared between groups. The learning curve for each system was evaluated using cumulative sum (CUSUM) analysis.

**Results:**

The 2D-navigation group had significantly shorter operative times (94.71 ± 14.38 vs. 105.40 ± 11.22 min) and lower radiation doses (81.0 ± 16.9 vs. 137.2 ± 43.0 µGy·m^2^) than the 3D-navigation group (*P* < 0.05). Screw placement accuracy did not differ significantly between groups (2D: 99.2% vs. 3D: 98.1%, *P* > 0.05). Both groups showed significant postoperative improvements in VAS and ODI scores, with no between-group differences at 1 week, 3 months, or final follow-up (all *P* > 0.05). No serious complications were observed in either group. CUSUM analysis indicated that proficiency was achieved earlier with 2D navigation (15 cases) than with 3D navigation (29 cases), suggesting a smoother learning curve for the 2D system.

**Conclusion:**

Compared with 3D navigation, 2D optical navigation reduced operative time and radiation exposure while maintaining comparable screw placement accuracy and clinical outcomes. With simpler operation and a shorter learning curve, 2D navigation-assisted PPSF may improve procedural efficiency and represents a promising alternative for minimally invasive treatment of thoracolumbar fractures.

## Background

Thoracolumbar fractures (TLFs) represent the most prevalent category of spinal injuries, particularly among individuals experiencing high-energy trauma or suffering from osteoporosis (1–4). While surgical intervention is essential for restoring stability and preventing neurological deterioration (5, 6), conventional open surgery is frequently associated with considerable blood loss, substantial tissue trauma, and prolonged recovery (7, 8). Consequently, Percutaneous Posterior Pedicle Screw Fixation (PPSF) has emerged as a preferred alternative, significantly minimizing surgical trauma and facilitating rapid functional recovery (7, 8).

Despite its advantages, PPSF presents significant technical challenges. The intricate anatomy of thoracic and lumbar pedicles exhibits substantial individual variation in morphology and orientation (9, 10). Under the restricted visualization of minimally invasive incisions, accurate screw placement becomes exceedingly difficult (11). Traditionally, surgeons rely on repeated 2D x-ray fluoroscopy. However, this method lacks spatial depth and necessitates frequent imaging, which not only significantly increases radiation exposure for both the patient and surgical team but also fails to completely eliminate the risk of screw malposition and subsequent neurovascular injury (12, 13).

To overcome these limitations, computer-assisted navigation， has been introduced to spine surgery (14, 15). Currently, the O-arm system coupled with optical navigation is considered the “gold standard,” offering intraoperative three-dimensional (3D) imaging that can reduce screw placement error rates to below 5% (16). However, the widespread adoption of 3D navigation is hindered by inherent drawbacks. These include a complex and time-consuming workflow necessitating repeated intraoperative scans, substantial radiation doses associated with 3D volumetric imaging, and a steep learning curve for instrument registration (17). Therefore, a navigation solution that balances high precision with workflow efficiency and reduced radiation remains a critical unmet need in spinal surgery.

To address these challenges, our center has applied an innovative PPSF technique utilizing a two-dimensional (2D) optical navigation system. Unlike O-arm based navigation, this system leverages standard fluoroscopic images for registration, potentially offering a streamlined workflow, reduced radiation exposure, and greater ease of use while maintaining real-time tracking capabilities. Preliminary observations suggest this technique enhances safety and precision compared to free-hand fluoroscopy. Nevertheless, there is a paucity of comparative literature evaluating whether this 2D navigation system can achieve clinical outcomes comparable to the established 3D O-arm system. In this study, we conducted a retrospective analysis to compare the accuracy, safety, and clinical effectiveness of 2D optical navigation-assisted PPSF against O-arm based 3D navigation in the treatment of TLFs.

## 1 Methods

### 1.1 Patient selection criteria

The study included patients who met the following criteria: (1) aged 18–60 years;(2) single-level traumatic fractures of the T11 to L2 vertebrae; (3) fractures classified as AO type A3 or A4; (4) intact bilateral pedicles at the fractured vertebrae; (5) no significant neurological impairment (Frankel grades D and E); (6) surgery performed within 2 weeks of injury; (7) no dislocation fractures; (8) availability of complete follow-up records and imaging data. Exclusion criteria included: (1) pathological fractures, such as those caused by tuberculosis or primary/metastatic tumors; (2) fractures involving or exceeding two vertebrae; (3) osteoporotic fractures; (4) a history of spinal surgical interventions or previous spinal trauma; (5) pedicle deformities or spinal deformities; (6) multiple organ system injuries; (7) pregnancy.

### 1.2 Patient population

This retrospective study included patients with TLFs who underwent PPSF at the Department of Orthopedics, Central Theater Command General Hospital of the People's Liberation Army, between January 2020 and February 2024. Based on predefined inclusion and exclusion criteria, 105 patients were eligible and enrolled. Patients were assigned to one of two groups according to the surgical method: the 2D-navigation group (PPSF assisted by a 2D optical positioning system) and the 3D-navigation group (PPSF guided by the O-arm 3D based navigation system). All procedures were conducted by two surgeons spine surgeons with similar training and clinical expertise. Because of the retrospective and non-randomized nature of this study, treatment allocation was based on the navigation modality used in routine clinical practice rather than random assignment. During the study period, both 2D optical navigation and O-arm-based 3D navigation were available as navigation-assisted options for PPSF. Before surgery, patients were informed of the available surgical options, and the final navigation modality was determined through shared decision-making between the patient and the treating surgeon. In addition, real-world factors, including operating-room scheduling, availability of the O-arm system, availability of the 2D optical navigation system, and the clinical workflow on the day of surgery, influenced the choice of navigation modality. The unequal distribution between the 2D-navigation group and the 3D-navigation group therefore reflected routine clinical practice and equipment availability rather than a pre-specified allocation ratio.

The study was approved by the Ethics Committee of the Central Theater Command General Hospital ((2025)066-01) and conducted in accordance with the principles of the Declaration of Helsinki. Written informed consent was obtained from all participants prior to enrollment.

### 1.3 Surgical technique

**Figure 1:**
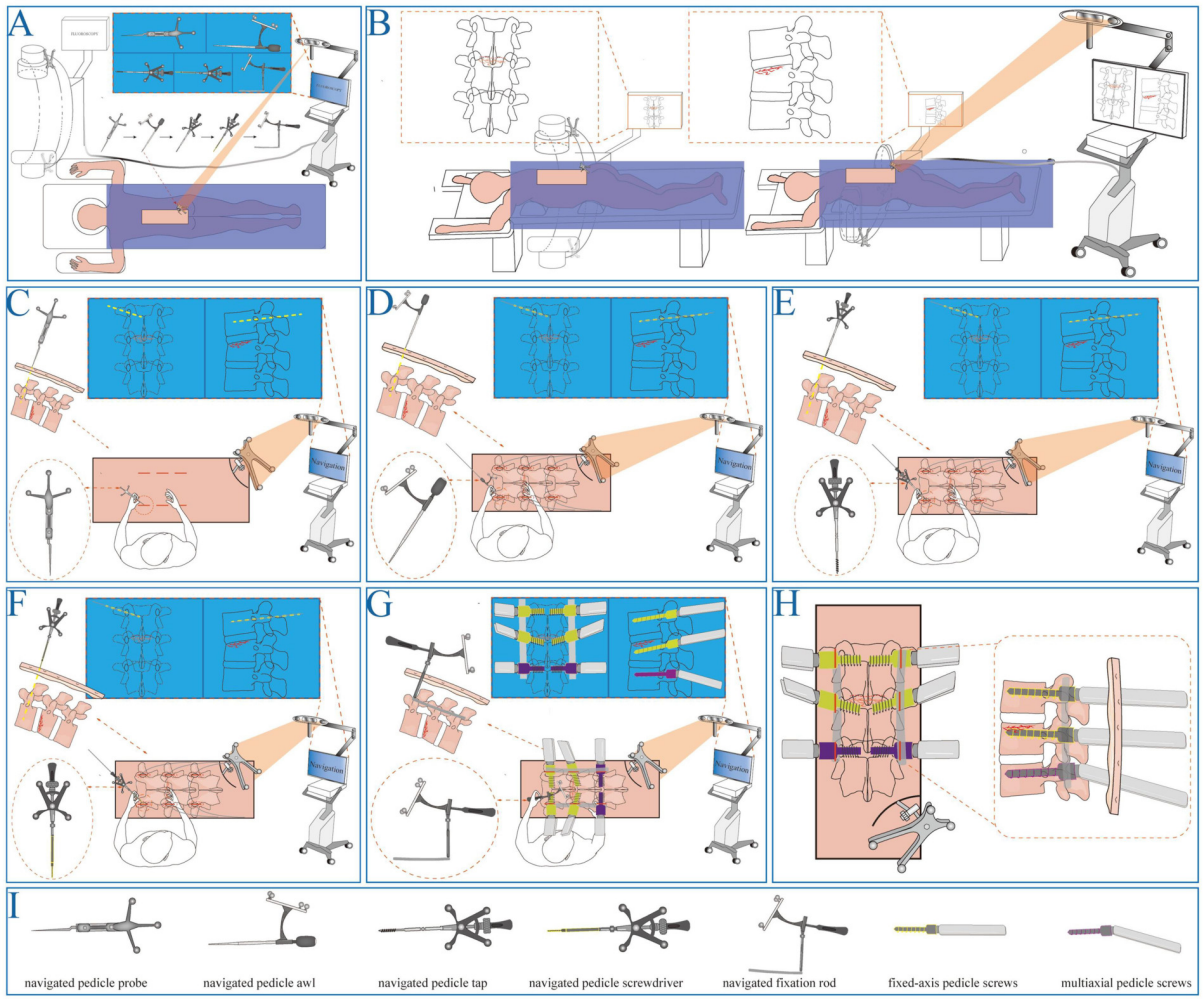
Surgical procedure of pedicle screw fixation under 2D optical navigation. **(A)** Instrument registration after reference frame fixation. **(B)** Target vertebra localization with C-arm and image reconstruction. **(C)** Incision planning using navigated pedicle probe. **(D–F)** Sequential pedicle canal preparation and screw insertion with navigated awl, tap, and screwdriver. **(G)** Placement of fixation rod according to physiological curvature. **(H)** Final tightening to restore alignment and vertebral height. **(I)** Navigation-assisted instruments used in the procedure.

The 2D optical navigation system used in the 2D-navigation group was the Boshikang/ZETNa system manufactured by Chongqing Boshikang Technology Co., Ltd. (Chongqing, China). This system uses an active–passive dual-mode optical tracking modality to track the patient reference frame and navigated surgical instruments in real time. The navigation workflow is based on a two-dimensional fluoroscopy-based registration strategy using a permanent calibration algorithm embedded in the system. First, the reference frame was fixed to the posterior superior iliac spine, and the surgical instruments were registered with the assistance of the scrub nurse (Figure 1A). The target vertebra was accurately identified using a C-arm, and intraoperative anteroposterior and sagittal images of the target vertebra were reconstructed and transmitted to the 2D optical navigation system (Figure 1B). A navigated pedicle probe was employed to ascertain the precise location for the surgical incision on the skin surface (Figure 1C). Using the optimal trajectory displayed on the 2D optical navigation system monitor, the navigated pedicle awl was inserted into the vertebra parallel to the upper endplate (Figure 1D). Subsequently, a K-wire was then placed through each pedicle canal, and the pedicle awl was withdrawn. A navigated pedicle tap was then used to prepare an appropriate channel for the pedicle screw (Figure 1E). The pedicle screw was threaded over the K-wire and advanced through the pedicle into the vertebra, after which the K-wire was removed following the proper placement of the pedicle screw (Figure 1F). Finally, after determining the appropriate physiological curvature, a navigated rod of suitable length, as determined by the navigation system, was used to connect the pedicle screws (Figure 1G). The nuts on both the caudal and cranial screws were tightened to reestablish the normal physiological curvature of the thoracolumbar, while the nut on the middle fixed-axis screws was locked to restore the vertebral height of the injured vertebrae using the leverage principle (Figure 1H). All procedure was conducted under real-time guidance from the optical navigation system monitor.

The internal fixation stabilization system was provided by Shandong Weigao, a company based in China. The 2D optical navigation system is compatible with intraoperative imaging devices, such as C-arms (Siemens, Munich, Germany) and O-arm™ (Medtronic, Minneapolis, USA) surgical imaging systems. Postoperatively, all patients received prophylactic antibiotics for 2 days, and the sterile dressing on the incision site was changed every two days until suture removal. Patients were encouraged to initiate physical activities with the support of a brace; however, strenuous and heavy activities were restricted for up to three months following surgery.

### 1.4 Clinical assessment

We collected demographic data on the patients, including gender, age, body mass index (BMI), cause of injury, fracture level, AO fracture classification, the Thoracolumbar Spine Injury Classification System (TLICS) score, Load Sharing Classification (LSC) score, and follow-up duration. Perioperative evaluation metrics included surgical incision length, operative duration, screw insertion time per screw, intraoperative blood loss, intraoperative radiation exposure, and postoperative hospitalization duration. Clinical evaluation was conducted to assess patients' functional status before surgery and at follow-up visits (1 week, 3 months, and the latest follow-up) using the Visual Analogue Scale (VAS) and Oswestry Disability Index (ODI) questionnaires.

### 1.5 Radiological assessment

Postoperative CT images were anonymized and evaluated in a randomized order. The image assessors were blinded to patient identity, navigation modality, operative records, and clinical outcomes. Pedicle screw placement accuracy, cranial facet joint violation, and screw-depth percentage were independently assessed by two experienced spine surgeons who were not involved in the index operations. Interobserver agreement was evaluated before consensus. Weighted kappa statistics were used for the Gertzbein-Robbins and Babu classifications, as both are ordinal categorical variables. The intraclass correlation coefficient was used to assess interobserver agreement for screw-depth percentage. Any disagreement between the two observers was resolved by consensus; if consensus could not be reached, a third senior spine surgeon was consulted for final adjudication. Imaging evaluation metrics were used to assess the accuracy of pedicle screw placement, the degree of cranial facet joint invasion, and the percentage of screw depth using axial CT images. Pedicle screw placement accuracy was assessed using the Gertzbein-Robbins grading system, classified as follows: Grade 0 indicates complete containment within the pedicle cortex; Grade 1 denotes penetration of less than 2 mm into the pedicle cortex; Grade 2 refers to penetration between 2 and 4 mm; and Grade 3 indicates penetration exceeding 4 mm. Screw placement accuracy was determined by dividing the number of Grade 0 and Grade 1 screws by the total number of screws, then multiplying by 100%. Cranial facet joint invasion was assessed according to the classification system established by Babu et al. Pedicle screw penetration into the cranial facet joint was classified as follows: Grade 0 indicates no contact with the cranial facet joint; Grade 1 indicates screw contact with the outer margin of the facet joint without capsule penetration; Grade 2 denotes penetration of the joint capsule by less than 1 mm; and Grade 3 indicates penetration of the joint capsule by 1 mm or more. The screw depth percentage was defined as the ratio of screw length in the axial plane to the vertebral body's anteroposterior diameter.

### 1.6 Learning curve analysis

In this study, the Cumulative Sum (CUSUM) analysis method was used to assess the learning curves of two surgical technical and pedicle screw placement, with the evaluation metrics being the surgical time and screw insertion time (18). First, the cases were arranged in chronological order based on the surgery date. The CUSUM value for the first case was determined by subtracting the average surgical time of all cases from the surgical time of that case. For the second case, the CUSUM value was calculated as the difference between the surgical time of the second case and the average surgical time of all cases, plus the CUSUM value of the first case. This process was repeated until the CUSUM value for the final case reached zero. To further analyze the learning curve, the CUSUM values were used for polynomial curve fitting to calculate the goodness of fit of the model. Then, by observing changes in the slope of the derivative of the fitted curve (i.e., the point where the curve transitions from an upward trend to a downward trend), critical points were identified, which allowed for the division of the learning phase and the proficiency phase. The learning curve for pedicle screw placement was calculated using the same method.

### 1.7 Statistical analyses

Statistical analysis was conducted with SPSS 27.0 (SPSS Inc., Chicago, IL, USA). Continuous data are expressed as mean ± standard deviation, while categorical data are shown as frequencies and percentages. The paired samples *t*-test, Mann–Whitney *U* test, Fisher's exact test, and chi-square test were employed to evaluate differences between the groups. A *P*-value <0.05 was deemed statistically significant.

## 2 Results

### 2.1 Demographic characteristics

A total of 105 patients meeting the inclusion criteria were analyzed, comprising 42 patients in the 2D-navigation group and 63 in the 3D-navigation group. The baseline characteristics, including age, gender, BMI, injury mechanism, and fracture classification, were well-balanced between the two cohorts. Specifically, the majority of fractures occurred at the thoracolumbar junction (T12 and L1), and falls from height were the leading cause of injury. No statistically significant differences were found in preoperative TLICS or LSC scores (*P* > 0.05), indicating comparable baseline injury severity (Table 1).

**Table 1:** Comparison of demographic characteristics between the two groups.

	2D group (*n* = 42)	3D group (*n* = 63)	*p* value
Age (years)	36.6 ± 8.3	35.1 ± 11.3	0.32
Sex(male)%	27 (64.3%)	39 (61.9%)	0.97
BMI	23.3 ± 1.4	23.5 ± 1.4	0.61
Causes of injury			0.92
Fall from height	15 (35.7%)	21 (33.3%)	
Traffic accidents	19 (45.2%)	31 (49.2%)	
Other	8 (19.1%)	11 (17.5%)	
Fracture level			0.76
T11	2 (4.8%)	2 (3.2%)	
T12	12 (28.6%)	19 (30.2%)	
L1	18 (42.9%)	22 (34.9%)	
L2	10 (23.8%)	20 (31.7%)	
AO classification			0.75
A3	32 (76.2%)	45 (71.4%)	
A4	10 (23.8%)	18 (28.6%)	
TLICS score			0.83
4	11 (26.2%)	19 (30.2%)	
5	31 (73.8%)	44 (69.8%)	
LSC score			0.98
3	3 (7.1%)	4 (6.3%)	
4	5 (11.9%)	9 (14.3%)	
5	13 (31.0%)	22 (34.9%)	
6	14 (33.3%)	18 (28.6%)	
7	7 (16.7%)	10 (15.9%)	
Duration of follow-up (months)	13.7 ± 1.4	13.4 ± 1.1	0.29

2D group, 2D-navigation system group; 3D group, 3D-navigation group; BMI, Body mass index. Data are presented as the mean ± standard deviation, and categorical variables are presented as frequencies and percentages. *P* values <0.05 represent significance.

### 2.2 Perioperative outcomes

Perioperative metrics are detailed in Table 2. The 2D-navigation group demonstrated a significantly shorter total surgical duration compared to the 3D-navigation group (94.71 ± 14.38 min vs. 105.40 ± 11.22 min, *P* < 0.01). Furthermore, intraoperative radiation exposure, measured by Dose Area Product (DAP), was markedly lower in the 2D-navigation group (81.0 ± 16.9 µGy.m^2^) than in the 3D-navigation group (137.2 ± 43.0 µGy.m^2^, *P* < 0.001). Conversely, no significant intergroup differences were observed regarding incision length, screw insertion time per screw, intraoperative blood loss, or length of hospital stay (*P* > 0.05).

**Table 2:** Comparison of the intraoperative and postoperative variables between the two groups.

	2D group (*n* = 42)	3D group (*n* = 63)	*p* value
Incision length(cm)	8.4 ± 1.1	8.6 ± 0.9	0.26
Surgical duration(min)	94.71 ± 14.38	105.40 ± 11.22	<0.01
Placement time/screw	6.2 ± 1.2	6.5 ± 1.3	0.37
Intraoperative blood loss(mL)	56.1 ± 15.1	59.5 ± 16.5	0.28
DAP(uGy*m^2^)	81.0 ± 16.9	137.2 ± 43.0	<0.001
Postoperative hospital stay(day)	8.9 ± 2.2	9.2 ± 1.9	0.46

2D group, 2D-navigation system group; 3D group, 3D-navigation group; DAP: Dose Area Product. *P* values <0.05 represent significance.

### 2.3 Learning curve comparison between groups

**Figure 2:**
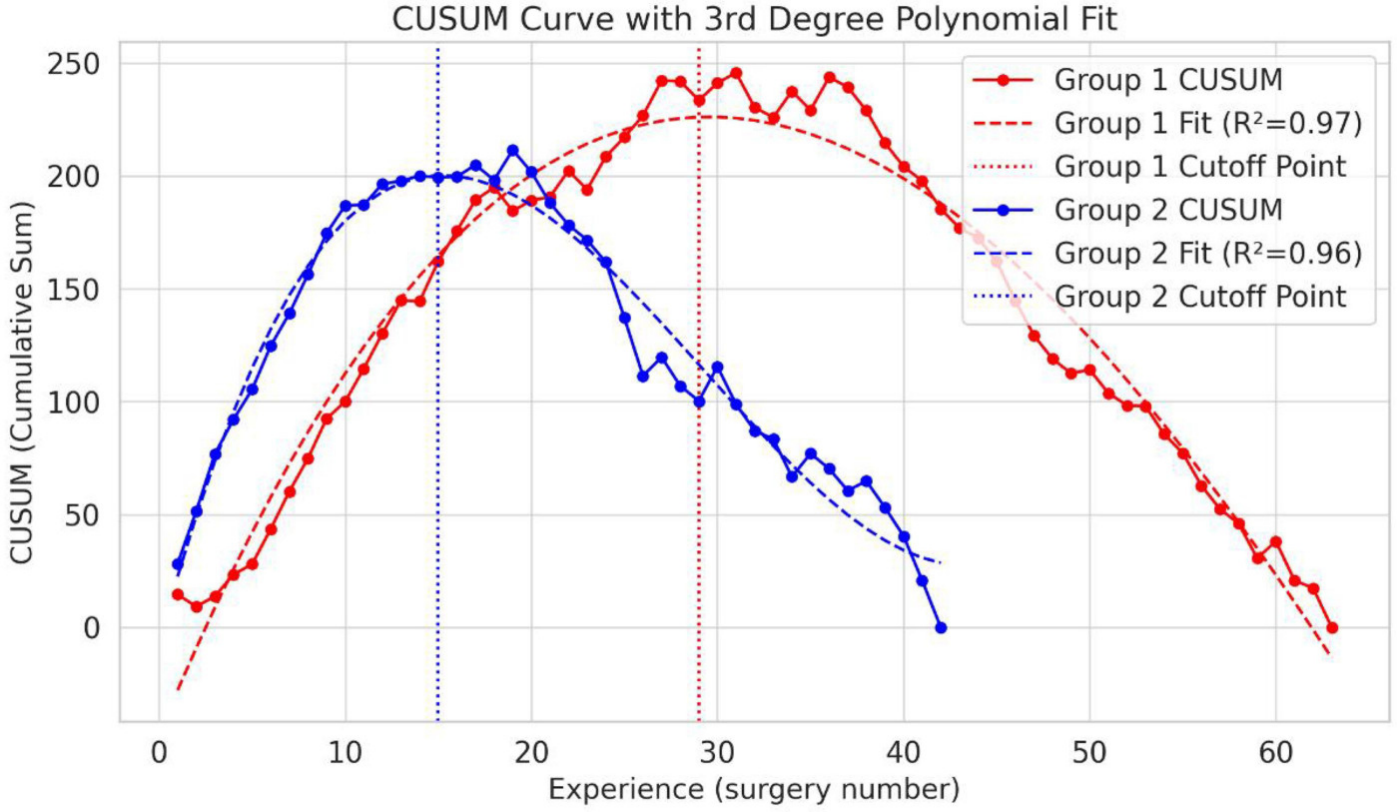
CUSUM, cumulative sum control chart. CUSUM learning curves of operative duration for the 2D- and 3D-navigation groups. The learning plateau was reached after 15 cases in the 2D-navigation group and after 29 cases in the 3D-navigation group.

The fitted curves demonstrated a good fit for both groups (*R*^2^ > 0.95). When the 3D navigation group's surgical cases reached 29, the slope of the fitted curve shifted from positive to negative, indicating the proficiency threshold. Similarly, for the 2D navigation group, the slope changed at 15 cases, suggesting that the learning curve plateaued earlier in this group (Figure 2).

### 2.4 Radiologic outcomes

Interobserver agreement was almost perfect for the Gertzbein–Robbins classification (overall weighted *κ* = 0.935, 95% CI: 0.894–0.969) and the Babu classification (overall weighted *κ* = 0.911, 95% CI: 0.848–0.950). The reliability of screw-depth percentage measurement was excellent (overall ICC = 0.954, 95% CI: 0.946–0.960). Screw placement accuracy was high in both cohorts. In the 2D-navigation group, 99.2% (250/252) of screws were clinically acceptable (Grade 0 and 1), compared to 98.1% (371/378) in the 3D-navigation group; this difference was not statistically significant (*P* = 0.72). Similarly, the rates of facet joint violation were comparable, with 89.3% of screws in the 2D group and 88.4% in the 3D group showing no violation (Grade 0) (*P* = 0.80). Screw depth percentages were also similar between groups (79.2 ± 4.9% vs. 78.2 ± 6.2%, *P* = 0.36) (Table 3).

**Table 3:** Comparison of radiological parameters between the two groups.

	2D group (*n* = 252)	3D group (*n* = 378)	*p* value
Accuracy of screw placement (*n*, %)			0.72
Grade 0	221 (87.7)	326 (86.2)	
Grade 1	29 (11.5)	45 (11.9)	
Grade 2	2 (0.8)	7 (1.9)	
Grade 3	0	0	
facet joint violation rate (*n*, %)			0.80
Grade 0	225 (89.3)	334 (88.4)	
Grade 1	23 (9.1)	39 (10.3)	
Grade 2	4 (1.6)	5 (1.3)	
Grade 3	0	0	
Screw depth (%)	79.2 ± 4.9	78.2 ± 6.2	0.36

2D group, 2D-navigation system group; 3D group, 3D-navigation group; *P* values <0.05 represent significance.

### 2.5 Comparison of the clinical and functional recovery outcomes

**Figure 3:**
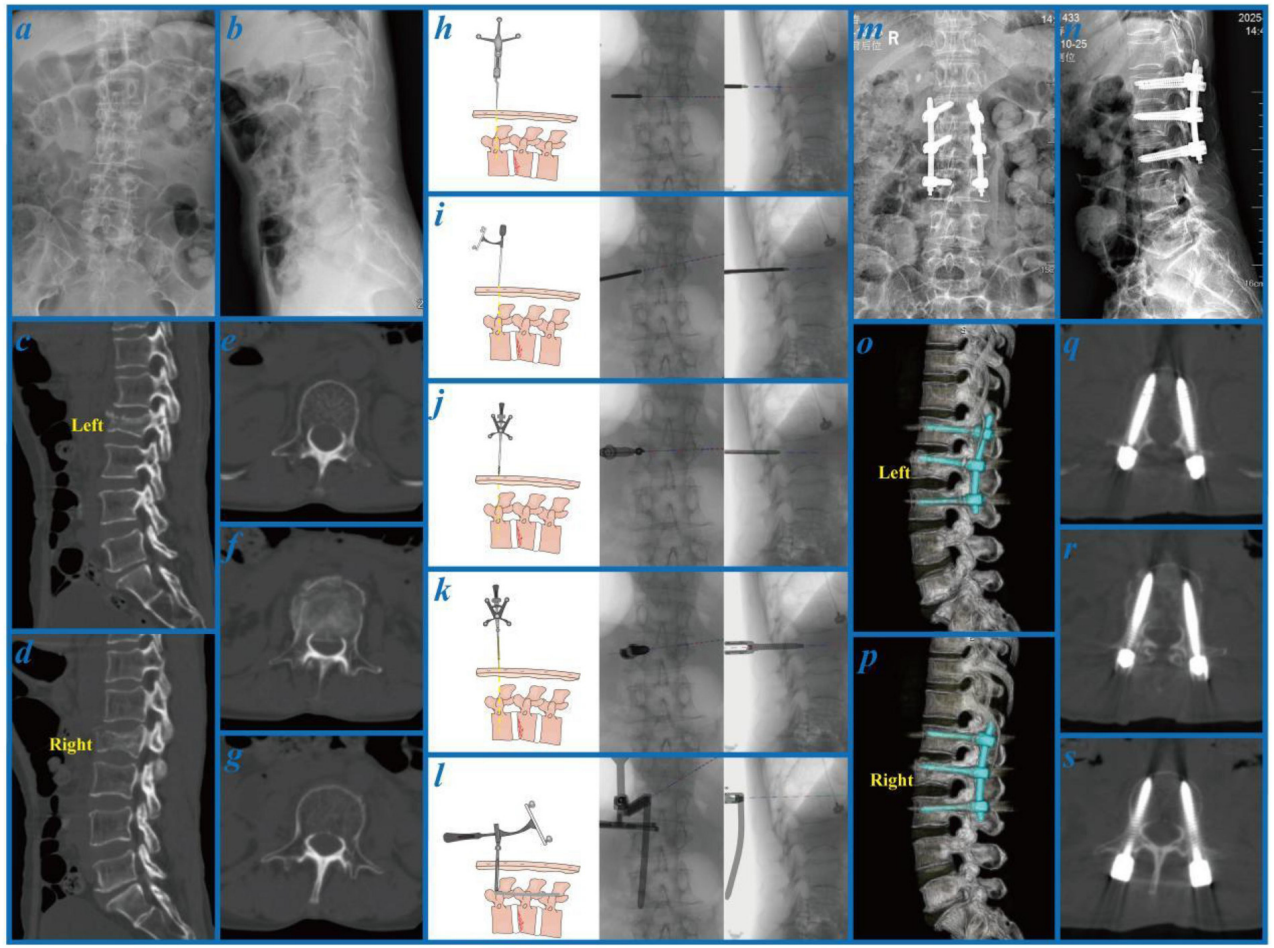
A 42-year-old female patient with an L2 burst fracture treated with PPSF under 2D navigation. **(a,b)** Preoperative anteroposterior and lateral radiographs showing the L2 burst fracture. **(c–g)** Preoperative sagittal and axial CT reconstructions demonstrating the L2 burst fracture with intact pedicles. **(h)** Navigated pedicle probe used to identify the optimal entry point and skin incision. **(i)** Navigated pedicle awl advanced into the pedicle and vertebral body along the planned trajectory. **(j)** Navigated pedicle tap employed to prepare the screw channel. **(k)** Under navigation guidance, a pedicle screw was inserted into the vertebral body using a navigated screwdriver. **(l)** A fixation rod of appropriate length was selected and positioned under navigation guidance. **(m,n)** Postoperative anteroposterior and lateral radiographs confirming satisfactory screw fixation. **(o–s)** Postoperative sagittal and axial CT images demonstrating accurate screw placement and adequate restoration of vertebral height.

Long-term clinical efficacy was assessed using VAS and ODI scores (Table 4). Preoperatively, both groups exhibited similar pain levels and functional disability (*P* > 0.05). Postoperatively, patients in both groups experienced significant relief in pain and improvement in function compared to baseline (*P* < 0.05). However, intergroup comparisons at 1 week, 3 months, and the final follow-up revealed no statistically significant differences in VAS or ODI scores (*P* > 0.05), suggesting that both navigation techniques yielded equivalent clinical recovery. No major complications, such as neurovascular injury, deep infection, or implant failure, were recorded in either group throughout the follow-up period. Representative cases from the 2D-navigation group are presented in Figure 3.

**Table 4:** Comparison of the clinical and functional recovery outcomes.

	2D group	3D group	*p* value
VAS score			
Pre-operation	7.2 ± 0.8	7.3 ± 0.8	0.67
1 week postoperatively	2.4 ± 0.6	2.4 ± 0.5	0.94
3 months postoperatively	1.3 ± 0.4	1.4 ± 0.4	0.26
Last follow-up	0.5 ± 0.3	0.6 ± 0.3	0.12
ODI score (%)			
Pre-operation	81.7 ± 8.2	83.5 ± 6.4	0.21
1 week postoperatively	33.2 ± 8.2	34.8 ± 5.7	0.18
3 months after surgery	14.0 ± 4.5	14.7 ± 4.8	0.45
Last follow-up	5.1 ± 2.4	5.3 ± 2.6	0.77

2D group, 2D-navigation system group; 3D group, 3D-navigation group; *P* values <0.05 represent significance.

## 3 Discussion

In this retrospective study, we applied a 2D optical navigation system to assist PPSF for TLFs. Notably, the system required only a single intraoperative anteroposterior and lateral fluoroscopic acquisition to complete image transfer and instrument registration. Once registration was completed, each procedural step—including incision planning, pedicle cannulation, tapping, screw insertion, and rod placement—could be performed under continuous navigation guidance. In this context, the proposed workflow supports an end-to-end navigated minimally invasive procedure and is designed to enhance both safety and placement precision. Consistent with this rationale, our results indicate that, relative to 3D navigation–assisted PPSF, 2D navigation–assisted PPSF reduced radiation exposure and shortened operative time while maintaining comparable screw placement accuracy and clinical outcomes.

Navigation systems can be classified by their tracking principle (e.g., optical, electromagnetic, and ultrasonic), with optical navigation being the most widely used in operative settings (14, 19, 20). Optical navigation tracks instrument position via infrared-based detection and has been shown to improve pedicle screw accuracy and reduce malposition rates. For example, prior work has reported improvements in accuracy from approximately 75%–95% with navigation assistance (21). Nevertheless, the adoption of 3D navigation in routine PPSF remains limited by workflow burden and radiation considerations. Specifically, 3D navigation–assisted PPSF typically requires additional time for scanning, registration, and positioning, and it may not substantially shorten overall operative duration compared with conventional experience-based percutaneous techniques (21). Moreover, concerns have been raised that intraoperative 3D imaging increases radiation exposure (22), and some evidence indicates that CT-based intraoperative imaging can approach or exceed annual adult exposure recommendations in certain contexts (23). These constraints provided the clinical impetus for developing a 2D navigation–assisted PPSF workflow. Importantly, the current literature contains few comparative studies evaluating 2D navigation–assisted PPSF for TLFs, thereby justifying further investigation.

Consistent with the above premise, our findings suggest that 2D navigation–assisted PPSF improves workflow efficiency and reduces operative time compared with 3D navigation–assisted PPSF. Specifically, mean operative duration was 94.71 ± 14.38 min in the 2D group vs. 105.40 ± 11.22 min in the 3D group. This difference is plausibly explained by reduced registration and preparation demands and improved procedural convenience. First, the 2D navigation workflow relies on a single anteroposterior and lateral image set to complete image generation and registration; in our series, registration required approximately 2–3 min, compared with 7–8 min for the 3D system. This is consistent with prior reports indicating that 3D navigation registration commonly requires 8–10 min (21). In addition, the O-arm platform is relatively bulky and less maneuverable than a conventional C-arm, which can further prolong setup and intraoperative repositioning, particularly in constrained operating rooms. Second, because 3D navigation systems are more complex, they often require dedicated technical expertise for efficient intraoperative preparation, which may also contribute to longer setup times. Taken together, these factors support the inference that shorter preoperative registration is a primary driver of the reduced operative time observed with 2D navigation.

Beyond setup time, the 2D navigation system may better align with established PPSF operating habits. In conventional PPSF, surgeons typically depend on anteroposterior and lateral views to infer pedicle orientation, which may necessitate repeated puncture attempts when anatomical cues are ambiguous (24, 25). By enabling direct visualization of pedicle entry point and trajectory within the familiar 2D imaging framework, the system may facilitate more efficient cannulation while preserving the surgeon's conventional decision process. By contrast, 3D navigation provides richer information but requires continuous integration of axial, sagittal, and coronal views to determine trajectory, which can increase cognitive and procedural complexity. In addition, navigation-assisted estimation of rod length may further reduce operative delays. In prior practice, rods that were too long or too short often required replacement and intraoperative readjustment, thereby prolonging surgery (26). In the present workflow, rod selection is informed by the navigated relative position of cranial and caudal screws, which may reduce repeated adjustments and contribute to time savings.

Although shorter operative duration is commonly associated with reduced blood loss, no significant between-group difference in blood loss was observed. This finding is likely attributable to comparable minimally invasive approaches and incision lengths across groups, which would be expected to yield similar baseline tissue disruption and bleeding. Moreover, the overall low invasiveness of both procedures may have limited the detectability of modest differences in blood loss.

To further characterize technical adoption, we used cumulative sum (CUSUM) analysis of operative time and screw insertion time to examine learning curves. Both surgeons had received formal training in minimally invasive techniques and had comparable academic and clinical backgrounds; moreover, each surgeon performed both procedures. Under these conditions, the 2D navigation workflow reached a proficiency plateau earlier than the 3D workflow, with inflection points occurring at 15 and 29 cases, respectively. This pattern supports the inference that 2D navigation may be easier to learn and execute in routine practice. One plausible explanation is that 2D navigation requires interpretation of only two planes of information, thereby reducing the need to integrate multiple 3D datasets during time-sensitive steps such as pedicle cannulation. For surgeons with baseline experience, assessment of guidewire–pedicle relationships on anteroposterior and lateral views is often sufficient to judge safety, which may facilitate smoother execution. Furthermore, because the 2D workflow resembles traditional PPSF, it may reduce the cognitive disruption associated with adopting a new technique, thereby accelerating skill acquisition and reducing fatigue (27, 28). From this perspective, the perceived “intuitiveness” of the 2D workflow may contribute to fewer errors and more rapid consolidation of procedural steps.

Previous studies have primarily emphasized 3D navigation to enhance pedicle screw accuracy (29, 30).Recent studies comparing robotic-guided, intraoperative CT-navigation-guided, and fluoroscopy-guided pedicle screw placement have emphasized that screw accuracy should be interpreted together with radiation exposure, implant-related outcomes, and workflow feasibility (31, 32). In this context, our data indicate that 2D navigation can achieve screw placement outcomes comparable to those obtained with 3D navigation in this trauma cohort. However, our data indicate that 2D navigation can achieve screw placement outcomes comparable to those obtained with 3D navigation in this trauma cohort. This finding provides empirical support for the viewpoint that volumetric imaging may not be strictly necessary for accurate screw placement in selected routine cases. In practical terms, anteroposterior and lateral information can be sufficient to ensure that the trajectory remains within the pedicle corridor. For example, when the puncture needle slightly extends beyond the posterior vertebral body edge on the lateral view while remaining within the medial and lateral pedicle boundaries on the anteroposterior view, safe intrapedicular placement can still be maintained. Accordingly, our results suggest that, even without axial images, 2D navigation can achieve positioning accuracy that is clinically comparable to 3D navigation in appropriately selected patients.

Using postoperative axial CT and the Gertzbein–Robbins grading system, we found clinically acceptable screw placement (Grades 0–1) of 99.2% in the 2D group and 98.1% in the 3D group, with no significant difference, consistent with prior reports (33, 34). Screw depth was also similar between groups (79.2 ± 4.9% vs. 78.2 ± 6.2%). While greater depth may theoretically enhance pullout resistance and construct stability, the observed difference was small and did not translate into differential clinical outcomes in this series. Likewise, facet joint violation rates did not differ significantly, suggesting that 2D navigation can be as effective as 3D navigation in limiting iatrogenic facet injury. Because malposition may compromise facet capsules and ligaments and contribute to instability and adjacent segment degeneration (35, 36), the comparable facet outcomes further support the overall safety of the 2D approach in this population.

Radiation exposure remains a major concern in minimally invasive spine surgery and is closely related to both fluoroscopy frequency and the use of intraoperative 3D imaging. Prior reports suggest that C-arm–guided PPSF may require approximately 12–30 fluoroscopic acquisitions (14, 37), which can prolong procedures and increase cumulative exposure. Although 3D navigation improves volumetric visualization, a single intraoperative CT acquisition typically requires more time than obtaining standard anteroposterior and lateral views and may deliver substantially higher radiation than a single radiograph pair (38). In the present study, DAP was significantly lower in the 2D navigation group (81.0 ± 16.9 μGy·m^2^) than in the 3D navigation group (137.2 ± 43.0 μGy·m^2^). Thus, these data support the inference that 2D navigation may provide an advantageous balance between procedural efficiency and radiation reduction without compromising accuracy.

Finally, clinical recovery was evaluated using VAS and ODI scores. Both cohorts demonstrated significant postoperative improvements relative to baseline, with continued recovery at 3 months and at final follow-up. Importantly, intergroup differences in VAS and ODI at each follow-up time point were not significant, indicating that both navigation strategies achieved comparable clinical benefit in this cohort.

## 4 Limitations

This study has several limitations. First, its retrospective design entails a risk of selection bias, and the modest sample size underscores the need for multicenter, prospective, randomized controlled trials for external validation. Second, the investigation was conducted at a single institution, which may introduce homogeneity effects on outcomes. Third, by excluding patients with pedicle anatomical variations or spinal deformities, the study limits the generalizability of 2D navigation–guided PPSF to more complex anatomies.

## 5 Conclusion

In conclusion, the novel 2D optical navigation system represents a highly effective and safe alternative to O-arm-based 3D navigation for the treatment of single-level thoracolumbar fractures. This study demonstrates that the 2D modality achieves screw placement accuracy and clinical outcomes equivalent to the 3D system, while offering distinct advantages in terms of superior procedural efficiency, minimized radiation exposure, and a significantly shorter learning curve. These findings challenge the prevailing notion that volumetric 3D imaging is mandatory for accurate screw placement in routine trauma cases. Instead, they suggest that 2D navigation provides an optimal balance between surgical precision and operational simplicity. While 3D navigation remains indispensable for complex deformities, the 2D optical navigation system offers a promising, accessible, and surgeon-friendly solution for standard minimally invasive spine surgery, potentially facilitating the broader adoption of navigation technology in clinical practice.

## Data Availability

The raw data supporting the conclusions of this article will be made available by the authors, without undue reservation.
